# Posterior cingulate gyri metabolic alterations in HIV-positive patients with and without memory deficits

**DOI:** 10.1590/0100-3984.2019.0093

**Published:** 2020

**Authors:** Diogo G. Corrêa, Eelco van Duinkerken, Nicolle Zimmermann, Rochele P. Fonseca, Emerson L. Gasparetto

**Affiliations:** 1 Department of Radiology, Hospital Universitário Clementino Fraga Filho, Universidade Federal do Rio de Janeiro (UFRJ), Rio de Janeiro, RJ, Brazil.; 2 Clínica de Diagnóstico por Imagem (CDPI)/DASA, Rio de Janeiro, RJ, Brazil.; 3 Center for Epilepsy, Instituto Estadual do Cérebro Paulo Niemeyer, Rio de Janeiro, RJ, Brazil.; 4 Department of Medical Psychology, Amsterdam University Medical Centers, Free University, Amsterdam, the Netherlands.; 5 Department of Psychology, Pontifícia Universidade Católica do Rio Grande do Sul (PUCRS), Porto Alegre, RS, Brazil.

**Keywords:** HIV, Memory deficit, Posterior cingulate gyrus, Magnetic resonance spectroscopy, HIV, Déficit de memória, Giro do cíngulo posterior, Espectroscopia por ressonância magnética

## Abstract

**Objective:**

We aimed to evaluate whether human immunodeficiency virus (HIV)-positive patients with and without clinically significant memory deficits and healthy control participants differ on in vivo hydrogen-1 magnetic resonance spectroscopy (H-MRS) in the posterior cingulate gyri.

**Materials and Methods:**

In total, 21 HIV-positive patients with memory deficit (HIV+wMD) were compared with 15 HIV-positive patients without memory deficit (HIV+wOMD) and 22 sex-, age-, and education-matched control participants. Memory impairments were classified based on the participants’ performance on the Rey Auditory Verbal Learning Test. Short echo time (30 ms), single-voxel H-MRS was performed using a 1.5-T magnetic resonance scanner.

**Results:**

The HIV+wMD and HIV+wOMD groups had higher choline/creatine ratio in the posterior cingulate gyri than the control group. There were no significant metabolite ratio differences between the HIV+wMD and HIV+wOMD groups.

**Conclusion:**

HIV-positive patients with and without memory deficits had significantly higher choline/creatine ratios than controls in the posterior cingulate gyri, which may reflect cerebral inflammation, altered cell membrane metabolism, microgliosis, and/or astrocytosis.

## Abstract (pt)

**Objetivo:**

Nós avaliamos se os pacientes HIV-positivos com e sem déficits de memória clinicamente significativos e controles saudáveis diferem na espectroscopia de prótons do giro do cíngulo posterior, por ressonância magnética (RM) cerebral.

**Materiais e Métodos:**

Vinte e um pacientes HIV-positivos com déficit de memória foram comparados com 15 pacientes HIV-positivos sem déficit de memória e 22 controles, pareados por sexo, idade e escolaridade. As deficiências de memória foram classificadas por meio do desempenho no Teste de Aprendizagem Auditivo-Verbal de Rey. A espectroscopia de prótons foi realizada com tempo de eco curto (30 ms), por voxel único, no giro do cíngulo posterior, utilizando aparelho de RM de 1,5 T.

**Resultados:**

Os pacientes HIV-positivos com e sem déficit de memória apresentaram aumento da relação colina/creatina no giro do cíngulo posterior, comparados aos controles. Não houve diferenças significativas nas relações metabólicas no grupo HIV-positivo com déficit de memória, em relação ao grupo de pacientes HIV-positivo sem déficit.

**Conclusão:**

Pacientes HIV-positivos com e sem déficits de memória apresentaram relações colina/creatina significativamente aumentadas em relação aos controles, no giro do cíngulo posterior, o que pode refletir inflamação cerebral, alteração do metabolismo da membrana celular, microgliose e/ou astrocitose.

## INTRODUCTION

The clinical manifestations of human immunodeficiency virus (HIV)-associated neurocognitive disorders (HAND) in the highly active antiretroviral (HAART) era differ substantially from classical descriptions of the dementia complex of acquired immunodeficiency syndrome^(1)^. Currently, memory and executive function deficits are most prominent, whereas in the pre-HAART era, deficits in motor skills, cognitive speed, and verbal fluency characterized patients with HAND^(2)^. The pattern of cognitive dysfunction currently reported in patients with HAND is more similar to other more common neurodegenerative disorders, such as Alzheimer’s dementia, than to classical HIV-associated dementia, which could create challenges in the differentiation of these diseases^(1,3)^.

Even with treatment, a high prevalence of HAND (58.5%) remains. In general, most cases of HAND are not associated with severity of the HIV infection, with patients with good infection control and a stable medical condition also presenting HAND^(4)^. However, even HIV-infected individuals with asymptomatic or mild cognitive impairment may be at increased risk of dementia and death^(3)^.

Additionally, with increasing life expectancy, the prevalence of neurocognitive impairment is expected to increase. This is due to an increase in age-related concomitant diseases, such as associated neurodegenerative diseases^(3,5)^. Some studies demonstrated interactions between aging and chronic HIV infection, with greater emphasis on possible interactions between HIV-infection and immunosenescence^(5,6)^.

Hydrogen-1 magnetic resonance spectroscopy (H-MRS) is a technique that provides noninvasive metabolic/biochemical information about tissues *in vivo*, without the need for biopsy or radioactive tracer injections. H-MRS of the posterior cingulate gyrus is extensively studied in Alzheimer’s dementia, showing lower N-acetylaspartate (NAA)/creatine (Cr) ratio and higher myoinositol (MI)/Cr and choline (Cho)/Cr ratios in patients with Alzheimer’s dementia than in controls^(7,8)^.

H-MRS has also been studied in HIV-positive patients, generally showing a decreased NAA/Cr ratio and increased Cho/Cr and MI/Cr ratios in several brain regions, including the medial temporal lobe, frontal lobe, and posterior cingulate gyrus^(9-18)^. However, with regard to the limbic system in the case of clinically significant memory deficits, posterior cingulate gyrus H-MRS quality is superior than medial temporal lobe H-MRS, due to the close proximity of the latter to the skull base causing susceptibility artifacts.

This study aimed to investigate and compare the potential differences in the posterior cingulate H-MRS measured metabolite concentrations in HIV-positive patients with clinically significant memory deficit and HIV-positive patients without memory deficit and controls.

## MATERIALS AND METHODS

### Participants

This study was approved by the Institutional Review Board of the Universidade Federal do Rio de Janeiro, and all participants provided informed consent for participation in the study. Between September 2009 and September 2015, 62 patients with HIV infection for at least 5 years, confirmed by enzyme-linked immunosorbent assay and Western Blot, were randomly selected from the infectious disease outpatient clinic of the Hospital Universitário Clementino Fraga Filho. The exclusion criteria included the following: reported use of illicit drugs in the previous year, neurological disorders, psychiatric illnesses, and magnetic resonance imaging (MRI) contraindications. The database also included 51 healthy controls.

For the current study, significant alterations on fluid-attenuated inversion recovery (FLAIR), such as encephalomalacia, tumors or advanced white matter disease, and not performing H-MRS were also considered as exclusion criteria. HIV-positive patients specifically had to use antiretroviral treatment, have an undetectable viral load in the serum (< 50 copies/µL), and have CD4+ lymphocytes > 200 cells/µL. HIV-positive patients were divided into two groups, according to the presence (HIV+wMD) or absence (HIV+wOMD) of clinically significant memory deficits, as assessed by the Rey Auditory Verbal Learning Test (RAVLT). All patients underwent extensive neuropsychological testing and MRI on the same day.

Considering the exclusion criteria, 26 HIV-positive patients were excluded. Of the 51 control participants initially selected, 29 were excluded because H-MRS was not performed in these patients. Of the 36 HIV-positive patients included, 21 had clinically significant memory deficits (58.3%) and 15 (41.7%) had not. As presented in Table 1, all groups were matched for age, sex, and educational level. The HIV-positive groups were comparable in terms of disease duration, CD4+ lymphocyte count, and had undetectable viral load in the blood. All the participants underwent the MRI examination in the same scanner and with the same protocol.

**Table 1:** Sociodemographic and clinical data of HIV-positive groups and control participants.

Variable	Groups	Mean	SD	F-stat	P
Age, years	Controls	46.36	9.82	1.91	0.15
	HIV+wMD	51.52	7.83		
	HIV+wOMD	48.73	7.81		
Years of known infection	Controls	—	—	0.93	0.34
	HIV+wMD	13.35	4.01		
Years of education	HIV+wOMD	11.62	6.34		
	Controls	11.05	5.39	0.78	0.46
	HIV+wMD	9.71	4.37		
Sex	HIV+wOMD	11.67	4.65		
	Controls	17 M/5 F	—	—	0.20
	HIV+wMD	18 M/3 F	—		
CD4+ T lymphocyte count at the time of MRI (cells/µL)	HIV+wOMD	9 M / 6 F	—		
	Controls	—	—	—	0.92
	HIV+wMD	620.28	406.20		
	HIV+wOMD	631.47	293.30		

SD, standard deviation; M, male; F, female; F-stat, F-statistics.

### Neuropsychological assessment

Memory scores were calculated based on the Brazilian version of the RAVLT^(19)^, transformed to Z-score (participant score minus normative mean divided by normative standard deviation). RAVLT is an oral verbal learning test that is administered using a 15-item list comprising five learning trials (A1, A2, A3, A4, and A5), one interference trial (B1), one trial directly after the interference trial (A6), and a test of delayed recall (A7). Participants were classified as impaired if they presented a result ≤ -1.5 on the composite score (mean Z-score A7 + mean Z-score B1 + mean Z-score A5)/3). The cutoff score of -1.5 standard deviation below the mean of normative data has been widely used to identify clinically relevant deficits^(20)^ and is consistent with the diagnostic criteria for mild cognitive impairment^(21)^.

Table 2 shows the neuropsychological tests used and the considered variables for each cognitive domain. All neuropsychological tests were performed by one neuropsychologist trained in cognitive evaluation, with 10 years of experience. We matched the groups based on other cognitive domains, to prevent confounding of our results by deficits in other cognitive functions (Table 3).

**Table 2:** Cognitive domains tested (excluding memory), with the tests used and the considered variables in each test.

Cognitive domains (excluding memory)	Neuropsychological tests	Variables considered
Attention	Bells Cancellation test	Omissions (time 1)
	Hayling test and Trail Making test	Errors (part A)
	Wechsler Adult Intelligence Scale-III	Digits and letter-number sequencing tasks
Executive functions	Stroop test	Color-word page, interference score
	Trail Making test	Time (part B), errors (part B), time B–time A, time B/time A
	Hayling test	Errors part B, time B–time A
Sensory-perceptual and motor skills domains	Brazilian Brief Neuropsychological Assessment Battery (NEUPSILIN)	Constructive praxis task
Processing speed	Bells Cancellation test	Time 1
	Hayling test and Trail Making test	Times (part A)
Verbal language	Montreal Communication Evaluation Battery	Semantic and phonemic verbal fluency tasks

**Table 3:** Comparative analysis among groups on cognitive domains Z-scores.

Cognitive domains	Groups	Mean Z-score	SD	F-stat	*P*
Episodic memory	Controls	–0.49	0.68		0.000
	HIV+wMD	–2.36	0.65		
	HIV+wOMD	–0.50	0.59		
Attention/working memory	Controls	–0.39	0.83	0.66	0.57
	HIV+wMD	–0.49	0.69		
	HIV+wOMD	–0.20	0.61		
Executive functions	Controls	–0.77	1.33	0.15	0.85
	HIV+wMD	–0.63	0.93		
	HIV+wOMD	–0.57	1.05		
Sensory-perceptual and motor skills domains	Controls	–0.59	1.01	0.89	0.41
	HIV+wMD	–0.87	1.31		
	HIV+wOMD	–1.06	1.06		
Processing speed	Controls	–1.90	3.41	1.19	0.31
	HIV+wMD	–1.05	0.99		
	HIV+wOMD	–1.02	1.28		
Verbal language	Controls	–0.58	0.64	0.18	0.83
	HIV+wMD	–0.51	0.76		
	HIV+wOMD	–0.67	0.88		

SD, standard deviation; F-stat, F-statistics.

### MRI acquisition

**Figure 1:**
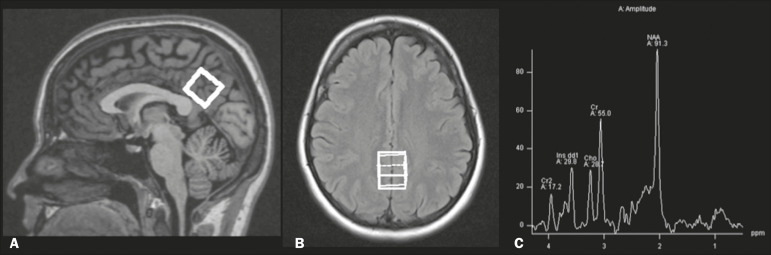
The H-MRS voxel was positioned in a region comprising parts of the right and left posterior cingulate gyri and the inferior precuneus gyri, using the midsagittal T1-weighted imaging (**A**) and axial FLAIR (**B**). **C:** Example of the H-MRS curve obtained in one of the participants.

MRI was performed using a 1.5-T scanner (Avanto, Siemens Healthcare, Erlangen, Germany) using an eight-channel phased-array head coil. The MRI protocol included the following: axial FLAIR (repetition time [TR] = 9000 ms; echo time [TE] = 83 ms; field of view [FOV] = 230 mm; matrix = 244 × 256; section thickness = 4.5 mm with a 10% gap; flip angle = 180°; inversion time = 2500 ms) and sagittal T1-3D magnetization-prepared rapid acquisition with gradient echo (MPRAGE)-weighted image (TR = 2730 ms; TE = 3.26 ms; inversion time = 1000 ms; FOV = 256 mm; matrix = 192 × 256; section thickness = 1.3 mm; flip angle = 7°; voxel size = 1.3 × 1.0 × 1.3 mm). The H-MRS was performed using an automated single-voxel package, through a point-resolved spectroscopy pulse sequence (TR = 1500 ms; TE = 30 ms; bandwidth = 1000; averages = 128). A voxel of 8 cm^3^ (2 cm × 2 cm × 2 cm) was positioned in a region comprising parts of the right and left posterior cingulate gyri and the inferior precuneus gyrus (Figure 1). Participants’ heads were stabilized with tape across the forehead and padding around the sides. The spectra were imported to a Leonardo workstation (Siemens), where dedicated manufacturer software for spectroscopy was used for peak identification and calculation of the metabolic ratios. All MRI images were reviewed by an experienced neuroradiologist and were of good quality.

### Statistical analyses

Demographic data was analyzed using the Statistical Package for the Social Sciences version 20 (IBM Corp., Armonk, NY, USA), by analysis of variance (ANOVA) with post-hoc Bonferroni or two-sample t-test for normally distributed variables or chi-squared tests for categorical variables.

The ratios between the amplitudes of the main metabolic peaks obtained in the H-MRS curves were calculated for each participant. The following metabolite ratios were taken into consideration: NAA/Cr, MI/Cr, Cho/Cr, and NAA/Cho ratios. An ANOVA, with Fisher’s least significant difference, was used to compare the mean metabolite ratio between the groups: HIV+wMD versus controls; HIV+wOMD versus controls; and HIV+wMD versus HIV+wOMD. A *p* < 0.05 was considered statistically significant. Furthermore, we assessed the association between the metabolic ratios and the composite memory score, considering the HIV-positive participants only, using the Pearson correlation coefficient in the SPSS version 20.

## RESULTS

### HIV+wMD versus controls

**Figure 2:**
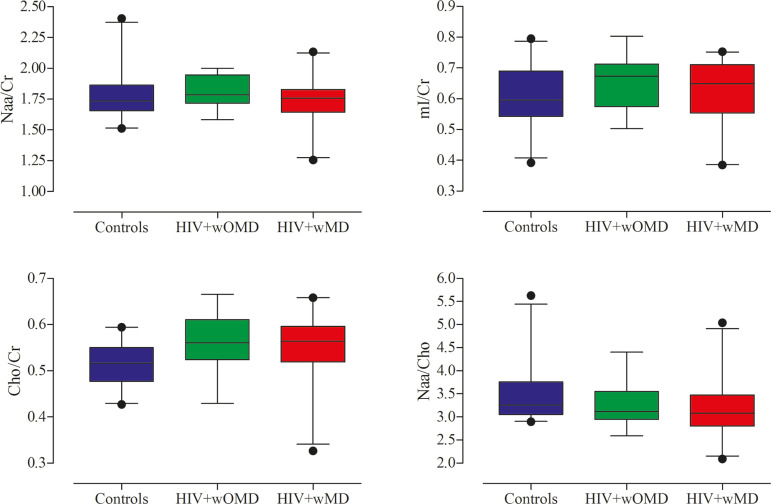
Box plots show the mean metabolite ratios in the posterior cingulate gyri of HIV-positive patients with and without memory deficit and controls. The metabolites ratios are associated with their amplitudes. Median values and 25th and 75th percentiles are shown in each box plot. Vertical bars represent the 5th and 95th percentiles. The circles represent outliers.

HIV+wMD presented a significantly higher mean Cho/Cr ratio than controls. There were no significant differences in the mean NAA/Cr, MI/Cr, and NAA/Cho ratios between HIV+wMD and controls (Figure 2 and Table 4).

**Table 4:** Mean metabolites ratios and standard deviation in each group and comparative analyses.

Metabolites ratios	Groups	Mean values	SD	F-stat	ANOVA (*p*)	Least significant difference	*P*
NAA/Cr	Controls	1.77	0.20	0.577	0.565		
	HIV+wMD	1.74	0.18				
	HIV+wOMD	1.80	0.13				
Cho/Cr	Controls	0.51	0.04	3.588	0.034	HIV+wMD versus Controls	0.031
	HIV+wMD	0.55	0.07			HIV+wOMD versus Controls	0.023
	HIV+wOMD	0.56	0.06			HIV+wMD versus HIV+wOMD	0.760
MI/Cr	Controls	0.61	0.09	1.068	0.351		
	HIV+wMD	0.61	0.11				
	HIV+wOMD	0.65	0.08				
Naa/Cho	Controls	3.48	0.64	1.563	0.219		
	HIV+wMD	3.18	0.57				
	HIV+wOMD	3.25	0.45				

### HIV+wOMD versus controls

HIV+wOMD also presented a significantly higher mean Cho/Cr ratio than controls. There were no significant differences in the mean NAA/Cr, MI/Cr, and NAA/Cho ratios between HIV+wOMD and controls (Figure 2 and Table 4).

### HIV+wMD versus HIV+wOMD

There were no significant differences in the mean metabolic ratios evaluated, in the posterior cingulate gyrus, between HIV+wMD and HIV+wOMD (Table 4).

### Association between the metabolic ratios and the memory score

Considering all HIV-positive participants as one group, there was no significant association between the metabolic ratios in the posterior cingulate gyri and the composite memory score (NAA/Cr: r = 0.212, *p* = 0.214; MI/Cr: r = 0.104, *p* = 0.545; Cho/Cr: r = 0.063, *p* = 0.714; and NAA/Cho: r = 0.067, *p* = 0.698).

## DISCUSSION

This study aimed to identify and compare the posterior cingulate metabolite differences between HIV+wMD versus HIV+wOMD and controls. We demonstrated that HIV+wMD and HIV+wOMD had higher Cho/Cr ratio in the posterior cingulate gyri than controls. However, the HIV-positive groups did not differed from each other with respect to H-MRS indices.

The main metabolites identified by H-MRS are NAA (peak at 2.02 ppm), Cho (3.22 ppm peak), Cr (peak at 3.02 ppm), and MI (peak at 3.56 ppm). NAA is a marker of neuronal and axonal integrity, and the reduction of its peak represents neuronal/axonal dysfunction or loss. Cho reflects cell membrane metabolism, and elevation of its peak is associated with increased cell population and increased cell membrane volume related to inflammation, demyelination, tumor, gliosis, and/or ischemia. MI is found in glial cells and is considered a marker of gliosis when found in high concentrations. Cr is a form of high-energy phosphate storage, which has relatively stable values in brain tissue and is used as a reference marker^(22,23)^.

Recent studies conducted in Brazil have highlighted the importance of imaging methods in the evaluation of diseases affecting the central nervous system^(24-29)^. Spectroscopy in the posterior cingulate gyrus is extensively studied in Alzheimer’s disease, in which previous studies showed lower NAA/Cr ratio in patients with Alzheimer’s disease than in individuals with mild cognitive impairment and controls. On the contrary, MI/Cr and Cho/Cr ratios were higher in patients with Alzheimer’s disease and with mild cognitive impairment than those of controls^(7,8)^.

H-MRS has also been studied in HIV-positive patients, generally showing a decreased NAA/Cr ratio and increased Cho/Cr and MI/Cr ratios in several regions of the brain, such as the basal ganglia^(9-11,30)^, frontal lobe^(9,10,12-14,30,31)^, supracallosal cortex^(15)^, posterior cingulate gyrus^(11,12,15,16)^, hippocampus^(32)^, caudate nucleus^(16)^, parietal-occipital white matter^(11)^, and areas with HIV encephalopathy^(17)^. However, these studies have not differentiated between patients with and without clinically significant memory deficits due to HIV. Although we found an increase in Cho/Cr ratio, we did not find any changes in NAA/Cr and MI/Cr ratios, which might be related to a difference in the inclusion criteria. Other studies have included patients with detectable viral load, CD4+ T lymphocytes < 200 cells/µL^(9,12)^, and dementia^(9-13,17)^ or did not analyze all major peaks in the spectroscopy curve^(14-18)^, whereas in this study, all patients had undetectable viral load and only had memory deficits. Moreover, the small sample size of this study may have contributed to the absence of differences in the other metabolic ratios.

Elevated Cho level may reflect cerebral inflammation, altered membrane metabolism, or HIV-infected monocytes’ responses, with microgliosis and/or astrocytosis^(33)^. Increased Cho is commonly observed among HIV-positive patients^(9-11,13,14,17,33,34)^. However, some studies failed to demonstrate this finding^(35,36)^. Although we found an increase in the Cho/Cr ratio in both HIV-positive groups in the current study, we were unable to differentiate the HIV-positive patients with memory deficit from those without this deficit. This may suggest that memory deficits were not associated with metabolite concentrations in the posterior cingulate gyrus. This hypothesis was corroborated by the lack of an association between the different metabolic ratios and the composite memory score in the HIV-positive participants in our study. Alternatively, this may be secondary to the clinical stability of the participants, reflected by a CD4+ T lymphocytes count > 200 cells/µL, undetectable viral load, absence of dementia, and stable use of HAART. Furthermore, increased Cho levels have been observed before decreased NAA levels, MRI abnormalities, and the onset of dementia were observed, which may therefore provide a useful marker for the early detection of brain injury associated with HIV infection^(9,14,34)^. Several studies have shown that HIV-positive patients with dementia present metabolic alterations, in relation to neuroasymptomatic HIV-positive patients, and even more accentuated alterations compared to healthy controls^(13,30)^. Furthermore, HAART may reverse, at least partially, some metabolic alterations^(9,12)^. For example, Stankoff et al.^(12)^ found that after HAART is initiated, MI/Cr and NAA/Cr ratios normalized or near-normalized in the frontal white matter of HIV-positive patients with and without cognitive impairment, whereas Cho/Cr ratios remained elevated in these groups. Our participants were dementia-free and under treatment and had no macrostructural MRI abnormalities; this might explain why only the Cho/Cr ratio was significantly increased.

This study has a few limitations. Considering that the study was designed as a cross-sectional study and all patients were on HAART, we could not examine the effects of treatment, lymphocyte CD4+ counts, or viral load on brain metabolites. Moreover, we assessed H-MRS of only one brain region, through a single-voxel technique. However, we studied the posterior cingulate gyri, a structure closely associated with memory and easily accessible to this technique. Additionally, the three groups were matched for age, educational level, sex, and other cognitive deficits. Although our sample was relatively small, which may have contributed to the fact that we did not find statistical differences in NAA/Cr, MI/Cr, and NAA/Cho ratios or between the two HIV-positive groups for the Cho/Cr ratio, we were able to show significant differences in Cho/Cr ratios of the posterior cingulate gyri between HIV-positive patients and controls. Our results show that even individuals on HAART with good infection control have metabolic changes in the brain, as do those who do not yet have clinically significant cognitive deficits.

In conclusion, HIV-positive patients with and without memory deficits had significantly higher Cho/Cr ratios than controls in the posterior cingulate gyri. However, posterior cingulate gyrus H-MRS could not differentiate HIV+wMD from HIV+wOMD.
